# Understanding the Virulence of *Staphylococcus pseudintermedius*: A Major Role of Pore-Forming Toxins

**DOI:** 10.3389/fcimb.2018.00221

**Published:** 2018-06-28

**Authors:** Yousef Maali, Cédric Badiou, Patrícia Martins-Simões, Elisabeth Hodille, Michele Bes, François Vandenesch, Gérard Lina, Alan Diot, Frederic Laurent, Sophie Trouillet-Assant

**Affiliations:** ^1^Centre International de Recherche en Infectiologie, Institut National de la Santé et de la Recherche Médicale, U1111, Centre National de la Recherche Scientifique, UMR5308, Université de Lyon 1, École Normale Supérieure de Lyon, Team “Pathogenesis of Staphylococcal Infections”, Lyon, France; ^2^Institut des Agents Infectieux, Hospices Civils de Lyon, Lyon, France; ^3^Centre National de Référence des Staphylocoques, Hospices Civils de Lyon, Lyon, France; ^4^Laboratoire Commun de Recherche Lyon Sud, Hospices Civils de Lyon-bioMérieux, Pierre Bénite, France

**Keywords:** *Staphylococcus pseudintermedius*, pore-forming toxins, phenol-soluble modulins, leukotoxin Luk-I, CXCR2 receptor, cytotoxicity, non-professional phagocytic cells, polymorphonuclear neutrophils

## Abstract

*Staphylococcus pseudintermedius* is responsible for severe and necrotizing infections in humans and dogs. Contrary to *S. aureus*, the pathophysiological mechanisms involved in this virulence are incompletely understood. We previously showed the intracellular cytotoxicity induced after internalization of *S. pseudintermedius*. Herein, we aimed to identify the virulence factors responsible for this cytotoxic activity. After addition of filtered *S. pseudintermedius* supernatants in culture cell media, MG63 cells, used as representative of non-professional phagocytic cells (NPPc), released a high level of LDH, indicating that the cytotoxicity was mainly mediated by secreted factors. Accordingly, we focused our attention on *S. pseudintermedius* toxins. *In silico* analysis found the presence of two PSMs (δ-toxin and PSMε) as well as Luk-I leukotoxin, the presence of which was confirmed by PCR in all clinical strains tested (*n* = 17). Recombinant Luk-I leukotoxin had no cytotoxic activity on NPPc but the ectopic expression of the CXCR2 receptor in U937 cells conferred cytotoxity to Luk-I. This is in agreement with the lack of Luk-I effect on NPPc and the previous report of Luk-I cytoxic activity on immune cells. Contrary to Luk-I, synthetic δ-toxin and PSMε had a strong cytotoxic activity on NPPc. The secretion of δ-toxin and PSMε at cytotoxic concentrations by *S. pseudintermedius* in culture supernatant was confirmed by HPLC-MS. In addition, the supplementation of such supernatants with human serum, known to inhibit PSM, induced a complete abolition of cytotoxicity which indicates that PSMs are the key players in the cytotoxic phenotype of NPPc. The results suggest that the severity of *S. pseudintermedius* infections is, at least in part, explained by a combined action of Luk-I that specifically targets immune cells expressing the CXCR2 receptor, and PSMs that disrupt cell membranes whatever the cell types. The present study strengthens the key role of PSMs in virulence of the different species belonging to *Staphylococcus* genus.

## Introduction

*Staphylococcus pseudintermedius* is a skin and mucous commensal bacterium in dogs, with carriage reaching more than 80% in some populations of healthy dogs (Rubin and Chirino-Trejo, 2011; Bannoehr and Guardabassi, 2012). This species is also the most frequent bacterial pathogen in clinical canine specimens mainly from skin wounds but also the ears, bones or post-surgical abscesses (Fazakerley et al., 2009; Miedzobrodzki et al., 2010; Bannoehr and Guardabassi, 2012). In addition to this virulence profile, methicillin-resistance in *S. pseudintermedius* (MRSP) has emerged worldwide over the two last decades and the treatment of *S. pseudintermedius* infections is now considered as a clinical challenge in veterinary medicine. In addition, *S. pseudintermedius* is increasingly isolated in human infections, mainly after contacts with dogs (Somayaji et al., 2016). Thus, since the first human case was described in 2006 (endocarditis occurring after the implantation of a cardioverter-defribrillator device Van Hoovels et al., 2006), sporadic community-acquired infections as well as nosocomial outbreaks involving *S. pseudintermedius* have been reported (Starlander et al., 2014; Somayaji et al., 2016).

Whether from canine or human origin, *S. pseudintermedius* infections are classically severe due to necrotizing processes, as illustrated in dermatitis and fasciitis in which a deep destruction of cells and tissues are observed (Weese et al., 2009). *S. pseudintermedius*, often described as the “dog's golden staph,” shares several features with *S. aureus*, notably the capacity to express a variety of virulence factors such as: (i) proteolytic enzymes including coagulase and protease (Garbacz et al., 2013), (ii) microbial surface components recognizing adhesive matrix molecules (MSCRAMMs), such as the staphylococcal protein A, that are host surface proteins and participate to the active tissue colonization and the evasion of the host immune system (Bannoehr et al., 2011, 2012; Balachandran et al., 2017), and (iii) toxins (Dziewanowska et al., 1996; Futagawa-Saito et al., 2004). Among the latter, pore-forming toxins (PFTs) have been extensively investigated for their ability to damage plasma membranes and lead to cell lysis (Prévost et al., 2001). Some of them induce cells death after the binding of the toxin with receptors on cell surface. This is the case for *S. aureus* bicomponent leukotoxins such as the Panton–Valentine leukocidin (PVL) or the leukotoxin ED (LukED) which act only on immune cells because of the interaction with cell receptors C5a or CXCR2, respectively (Reyes-Robles et al., 2013; Spaan et al., 2013). *S. pseudintermedius* produces a bicomponent leukotoxin, Luk-I, composed of the secreted LukS-I and LukF-I proteins that induce cell lysis. This activity has been reported only on PMNs and to date, no receptor has yet been identified (Prevost et al., 1995). Recently, *in vitro* studies underlined the highly virulent behavior of *S. pseudintermedius* on non-professional phagocytic cells (NPPc) such as canine keratinocytes, human epithelial cells and human osteoblasts, which are the most impacted cells during *S. pseudintermedius* infections (Pietrocola et al., 2015; Maali et al., 2016). Pietrocola et al. suggested that the *S. pseudintermedius* bicomponent leukotoxin Luk-I could also be involved in the cell death observed after internalization of *S. pseudintermedius* in NPPc (Pietrocola et al., 2015). To the best of our knowledge, this hypothesis has yet to be investigated. Phenol-soluble modulins (PSMs) represent another class of membrane damaging staphylococcal toxins. This class of amphipathic proteins has alpha-helical structure, and has a direct and receptor-independent action on the lipid bilayers of cells (Peschel and Otto, 2013). Various types of PSMs (α, β, δ-toxin…) have been described and are highly conserved among *S. aureus* or *Staphylococcus* non-*aureus* (SNA) strains (Cheung et al., 2014; Cameron et al., 2015; Da et al., 2017); whole genome sequencing analysis of *S. pseudintermedius* has revealed the presence of several PSMs: δ-toxin, PSMβ, and PSMε (Cheung et al., 2014).

Taking into consideration all of these elements, we sought to identify the virulence factors involved in the cytolytic effect on NPPc and immune cells related to severe and necrotizing *S. pseudintermedius* infections. Using *in vitro* cellular models (immune cells, NPPc, and transfected cells) and purified synthetic or recombinant toxins, bacterial culture supernatants with or without PSMs inhibitors; we identified the membrane receptor of Luk-I present on immune cells and demonstrated the specific *S. pseudintermedius* cytolytic activity of PSMε and δ-toxin on both NPPc and immune cells.

## Materials and methods

### Human ethics statement

This study was approved by the French South-East ethics committee (reference number 2013-018). In accordance with French legislation, written informed patient consent was not required for the use of the collected clinical isolates.

### Bacterial strains and culture media

A collection of 17 human and animal clinical *S. pseudintermedius* isolates was used. Characteristics of the isolates are summarized in Table 1. Identifications of all strains were confirmed using matrix-assisted laser desorption ionization time-of-flight mass spectrometry (MALDI-TOF-MS; VITEK® MS, bioMérieux, Marcy-L'Etoile, France) (Silva et al., 2015). The strains *S. aureus* LAC USA300 and its isogenic mutant *S. aureus* LAC Δ*psm* (α, β, δ), kindly provided by Frank Deleo (National Institutes of Health, Hamilton, MT, US) were used as positive and negative controls, respectively, for cytotoxicity experiments (Wang et al., 2007; Davido et al., 2016).

**Table 1:** Description of the strains used.

**Species**	**Strains**	**Origin**
*S. aureus*	LAC USA300 WT	Human blood
*S. aureus*	LAC USA300 Δ*psm (α,β,δ)*	Human blood
*S. pseudintermedius*	LMG 22219	Feline lung tissue
*S. pseudintermedius*	LMG 22220	Horse skin lesion
*S. pseudintermedius*	N900260	Human skin lesion
*S. pseudintermedius*	N930300	Human nasal carriage
*S. pseudintermedius*	N940453	Human blood culture
*S. pseudintermedius*	N950082	Human cerebrospinal fluid
*S. pseudintermedius*	LY19990344	Human skin lesion
*S. pseudintermedius*	ST20091777	Human pacemaker
*S. pseudintermedius*	ST20101529	Human bone biopsy
*S. pseudintermedius*	ST20112488	Human aspiration
*S. pseudintermedius*	ST20112499	Human blood culture
*S. pseudintermedius*	ST20120906	Human drain fluid
*S. pseudintermedius*	ST20121859	Dog skin pustule
*S. pseudintermedius*	ST20141177	Human hip prosthesis
*S. pseudintermedius*	ST20141277	Human leg abscess
*S. pseudintermedius*	ST20141366	Human nasal carriage
*S. pseudintermedius*	ST20141676	Human skin lesion

### Screening, diversity, and purification of *S. pseudintermedius* toxins

Screening for the presence of *lukF-I* and *lukS-I* genes encoding leukocidin Luk-I, *psm*ε that encodes PSMε, and *hld* that encodes δ-toxin was performed in the *S. pseudintermedius* isolates by PCR. Oligonucleotides and annealing temperatures are presented in the Supplementary Table 1. The resulting PCR products were sequenced (Biofidal, Lyon, France) and aligned using SeaView (Version 4.6.3; PRABI, Lyon, France) to investigate the genetic polymorphism of each gene (Gouy et al., 2010).

Purified toxins were then obtained by recombinant or synthetic production. For Luk-I, *lukS-I* and *lukF-I* genes were amplified by PCR using chromosomal DNA of *S. pseudintermedius* LMG 22219 using flanking primers, the sequences of which are presented in Supplementary Table 1. PCR products were codigested with *Bam*HI and *Sal*I enzymes (New England Biolabs, Ipswich, MA,US) then purified using the High Pure PCR Product Purification kit (Roche, Meylan, France) and ligated using T4 DNA Ligase (Promega, Madison, WI, USA) into the pQE-30 plasmid (Qiagen, Courtaboeuf, France). The resulting pQE-*lukS-I and* pQE-*lukF-I* plasmids were transformed into *E. coli* strain M15. Recombinant His-tagged toxins were expressed in LB medium and purified with Ni-nitrilotriacetic acid columns (Qiagen) before being dialyzed against phosphate-buffered saline (PBS). After protein purity was checked using SDS-PAGE and protein quantification performed using the Bradford method, LukS-I and LukF-I were combined and used for the cytotoxicity assays described below.

For PSMs, amino acid sequences corresponding to PSMε and the two variants of δ-toxin (variant ED99 and variant HKU10-03) were synthetized and purified to >90% by Genecust (Dudelange, Luxembourg) using high-performance liquid chromatography (HPLC).

### HPLC-MS analysis of PSMε and δ-toxin secreted in bacterial supernatants

Staphylococcal supernatants of tested strains were prepared by growing staphylococci bacteria in brain heart infusion (BHI) medium (bioMérieux) in a rotary shaker (190 rpm) at 37°C for 22 h, followed by centrifugation for 10 min at 3,000 × g. The filtered supernatants were stored at −80°C before being tested. For PSM quantification, bacterial supernatants were diluted 1:5 in methanol at 4°C, and incubated for 10 min at 4°C for cold precipitation to eliminate particulate matter. After centrifugation at 10,000 × g for 5 min, PSMε and δ-toxin (variant ED99 and variant HKU10-03) were quantified using a targeted approach by HPLC—mass spectrometry (HPLC-MS) in an Agilent® system as described previously (Hodille et al., 2016).

### Synthetic or recombinant *S. pseudintermedius* toxins cytotoxic assay

All cell culture reagents were obtained from Gibco (Paisley, United Kingdom). Human MG63 osteoblastic cells (LGC Standards, Teddington, UK), an NPPc cell line, were cultured in Dulbecco's modified Eagle's medium (DMEM) containing 2 mM L-glutamine and 25 mM HEPES and supplemented with 10% heat-inactivated fetal bovine serum (FBS) as well as 100 U/mL penicillin and streptomycin. Cells were passaged twice a week and were used in experiments up to passage 20 after being thawed from a stock culture.

U937 cells (human promyelocytic cell line) and U937 cells stably transfected with human C5a receptor (U937-C5aR), or with the CXCR2 (U937-CXCR2) kindly provided by Jos A. G. Van Strijp (Department of Medical Microbiology, Utrecht, Netherlands), were cultured at 37°C, 5% CO_2_ in Roswell Park Memorial Institute medium (RPMI) 1640 containing 10% heat-inactivated FBS (Kew et al., 1997).

Human polymorphonuclear neutrophils (PMNs) collected from the blood of healthy donors were isolated by density gradient centrifugation on Ficoll/Histopaque (Sigma-Aldrich) (Veldkamp et al., 2000). Viability of PMNs was determined by trypan blue exclusion and ranged from 95 to 99%. PMN were maintained at 37°C, 5% CO_2_ in RPMI 1640 containing 10% heat-inactivated FBS.

The cytotoxicity induced by the staphylococcal toxins was evaluated by measuring propidium iodide (PI) incorporation in to cells exposed to toxins using a fluorescence cell sorter (Spark®, TECAN, Zürich, Switzerland). Briefly, MG63 osteoblasts were seeded at 10^4^ cells per well on 96-well tissue culture plates (Falcon, Le Pont de Claix, France) in 100 μL of culture media. After 24 h, the cells were washed three times with 100 μL of PBS (Gibco). Immune cells (U937 and PMN) at 10^5^ cells per well were seeded in tissue culture plates the day of the experiment. All cells were incubated for 3 h at 37°C with toxins and PI (1.25 μg.mL^−1^). For the Luk-I toxin, 0.5 to 5 μg.mL^−1^ were tested after dilution in culture medium. For PSMε and the two δ-toxin variants, 0.1 to 100 μg.mL^−1^ were tested after dilution in DMEM (Flammier et al., 2016).

The negative control (Cneg) corresponded to the same experiment without toxin. The positive control (Cpos) was obtained using complete cell lysis solution (Triton X100 0.1%). As previously described, cell lysis due to toxins was expressed as percentages using the following formula: $\%=\frac{\text{Test}-\text{Cneg}}{\text{Cpos}-\text{Cneg}}\times 100$ (Hodille et al., 2016).

### *S. pseudintermedius* culture supernatants cytotoxicity assay

Because of the high autofluorescence of BHI medium, the cytotoxic effect of culture supernatant on NPPc could not be performed using IP incorporation. To circumvent this issue, cytotoxicity was evaluated by measuring Lactate dehydrogenase (LDH) release in the cell culture supernatant using a colorimetric method (Dimension Vista automated clinical chemistry analyzer, Siemens Healthcare Diagnostics, Tarrytown, NY, USA). In parallel, the cytotoxic effect of the supernatant was investigated by microscopy; after being fixed using formaldehyde 4% for 20 min, and stained 30 min using Giemsa stain, modified solution (Sigma-Aldrich) according to the manufacturer's instructions, cells were observed under a light microscope Leica DMi1 (Leica Microsystems, Wetzlar, Germany) at a magnification of × 20. The supernatant from cytotoxic *S. aureus* LAC USA300 strain was used as positive control, and sterile BHI medium was used as negative control.

Osteoblasts were seeded at 100,000 cells per well on 24-well tissue culture plates (Falcon) in 1 mL of culture media. After 24 h, the cells were washed twice with 1 mL of DMEM before the addition of filter-sterilized (0.22 μm) staphylococcal culture supernatants. Serum inhibition of PSM cytotoxic activity in supernatant was tested using pooled human serum obtained from healthy donors provided by the French national public blood service (*Etablissement Français du Sang*, Lyon, France). Filter-sterilized supernatants were mixed with or without 5% inactivated human serum for 10 min at room temperature (Surewaard et al., 2012). Then, NPPC (osteoblasts) were incubated for 4 h at 37°C, 5% CO_2_.

### Statistical analysis

Non-parametric statistical Mann-Whitney *U*-tests and B tests with a threshold of significance of 0.05 were used to determine statistically significant differences. All analyses were performed using XLSTAT software (Pearson Edition, developed by Addinsoft v.2014; New York, NY, US).

## Results

### Cytolytic activity of *S. pseudintermedius* supernatants

**Figure 1:**
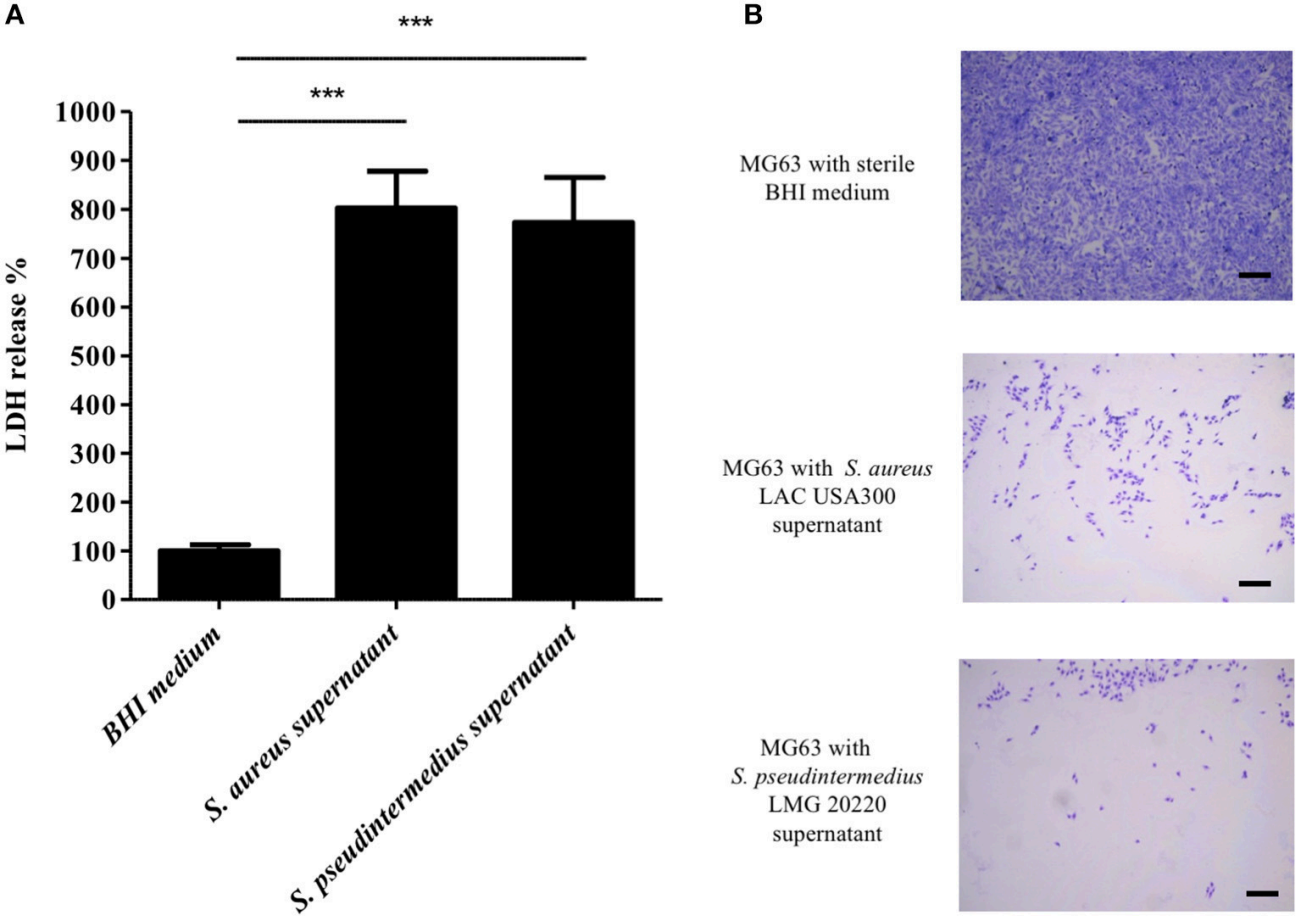
Effect of *S. pseudintermedius* supernantant on NPPc (MG63). MG63 cells were incubated with staphylococcal supernatants for 4 h at 37°C. **(A)** Quantification of LDH release, reflecting NPPc lysis by staphylococcal supernatant. All of the results are expressed as the percentages of the values obtained for the control (BHI medium alone, 100%). Bars represent means ± standard deviations derived from 3 experiments performed in triplicate. The difference in the LDH concentration in staphylococcal supernatants condition compared to BHI medium alone was evaluated using a one-tailed Mann-Whitney test with a α risk of 0.05 (^***^p < 0.001). **(B)** MG63 lysis after 4 h in contact with staphylococcal supernatant. The cells were stained with Giemsa, and observed for morphological changes by light microscopy at a magnification of ×20. Bars, 500 μm. LDH, Lactate dehydrogenase; NPPc, Non-professional phagocytic cells.

Knowing that the supernatant of *S. pseudintermedius* is able to induce the death of human PMNs (Riegel et al., 2011), we specifically performed an *in vitro* cytotoxic assay to evaluate its capacity to also kill non-immune cells, such as NPPc. First, cytolytic activity expressed as the means ± standard deviations quantified by LDH release from osteoblasts incubated with *S. pseudintermedius* LMG 20220 culture supernatant (773 ± 92%), was significantly higher than those observed in the control condition (100 ± 12%, *p* < 0.001; Figure 1A). Like *S. pseudintermedius, S. aureus* culture supernatant has a high cytotoxic effect on NPPc. Second, the microscopic observations indicated a high cytotoxic action of the supernatant from *S. pseudintermedius* LMG 20220 on MG63 cells, as demonstrated by the lysis area compared to the uniform cell layer observed in the control condition; the same phenotypic profile was observed for the hyper virulent strain *S. aureus* LAC USA300 (Figure 1B). These results demonstrate that the cytotoxicity induced by *S. pseudintermedius* is mediated by secreted virulence factors, which prompted us to investigate the specific bacterial factors involved, especially among staphylococcal toxins with cellular tropism.

### Luk-I toxin distribution and activity

**Figure 2:**
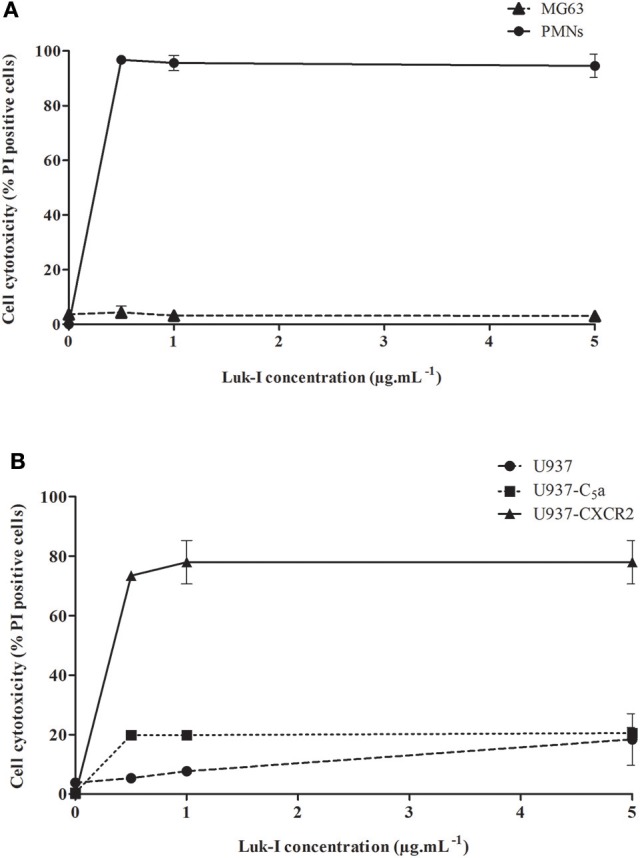
Cytolytic activity of *S. pseudintermedius* leukotoxin Luk-I. **(A)** Leukotoxin Luk-I toward NPPc (MG63) and immune cells (human PMNs). **(B)** Leukotoxin Luk-I activity on wild-type U937 cells and U937 cells expressing the C5a or CXCR2 receptors. Cells were incubated with increasing doses of recombinant Luk-I (from 0.5 to 5 μg.mL^−1^) for 3 h at 37°C. Cell death was measured by staining cells with propidium iodide (PI) and detected with a fluorescence cell sorter. The percentage of cell lysis was calculated as $\%=\frac{\text{Test}-\text{Tneg}}{\text{Tpos}-\text{Tneg}}\times 100$. The values represent the means ± standard deviations derived from one experiment performed in triplicate representative of two others. NPPc, Non-professional phagocytic cells; PMNs, Polymorphonuclear neutrophils; PI, Propidium iodide; U937-C5a, U937 cells transfected with C5a receptor; U937-CXCR2, U937 cells transfected with CXCR2 receptor.

PCR experiments found that all *S. pseudintermedius* isolates were positive for *lukF-I* / *lukS-I* genes. The use of recombinant Luk-I (combining LukS-I and LukF-I) allowed us to specifically analyze *in vitro* the cytotoxic capacity of this toxin. PMNs were used as control because the activity of Luk-I on these cells has been previously described (Prevost et al., 1995). As expected, a very high cytotoxic activity was observed for the PMNs cells, and this was found at the lowest concentration of recombinant Luk-I tested (0.5 μg.mL^−1^; Figure 2A). Conversely, the same toxin had no cytotoxic activity against NPPc for all tested concentrations (from 0.5 to 5 μg.mL^−1^). In order to identify the mechanism of this specific activity for PMNs, we tested the activity of toxins on wild-type U937 cells and transfected U937 cells that express the C5a or CXCR2 receptors; Luk-I had a high cytotoxic action only on cells that expressed the CXCR2 receptor (Figure 2B). This result suggests that the toxin Luk-I exhibits a tropism for myeloid cells expressing the CXCR2 receptor.

### Genetic characterization, distribution, and production of PSMε and δ-toxin in *S. pseudintermedius*

Due to the absence of activity for Luk-I on NPPc (MG63 cells), we speculated that PSMs could be the key virulence factors implicated in the *S. pseudintermedius* virulence observed on these cells. Two PSMs with cellular tropisms have been identified in *S. pseudintermedius* using *in silico* analysis based on the NCBI database: PSMε and the δ-toxin, for which two variants (ED99 and HKU10-03) have been described (Cheung et al., 2014). We therefore screened for the presence of these PSMs in the collection of clinical strains. Sequencing of the PCR products found the presence of *psm*ε and *hld* in all *S. pseudintermedius* isolates (*n* = 17). For δ-toxin, 14/17 isolates (82%) harbored the variant ED99 while 3/17 (18%) harbored the variant HKU10-03 (Table 2).

**Table 2:** Amino acid sequence of *S. pseudintermedius* phenol-soluble modulins (PSM).

**PSM name/annotated as**	**Amino acid sequence**	**Prevalence**
PSMε	MFIIDLIKKVIEFLKGLFGNK	17/17
δ-toxin (ED99)	MAADIISTIVEFVKLIAETVAKFIK	14/17
δ-toxin (HKU10-03)	MAADIISTIVEFVKLIAETVEKFIKK	3/17

To investigate the expression of these 3 PSMs, we quantified the level of their secretion in the supernatant of strains *S. pseudintermedius* LMG 20220 and *S. pseudintermedius* ST20112488, which respectively secrete δ-toxin variant ED99 and variant HKU10-03 according to the genetic profile (Supplementary Figure 1). Both PSMε and the two δ-toxin variants were detected and distinguished by HPLC-MS, and the mean ± SD concentrations in culture supernatants ranged from 22.4 ± 2.8 μg.mL^−1^ to 159.3 ± 83.9 μg.mL^−1^ (Table 3).

**Table 3:** Phenol-soluble modulins (PSM) concentrations in *S. pseudintermedius* supernatants.

***S. pseudintermedius* isolates**	**Concentrations (μg.mL^−1^)**		
	**PSM**ε	δ**-toxin** **(ED99)**	δ**-toxin** **(HKU10-03)**
*S. pseudintermedius* ST20112488	35.54 ± 4.74	ND	102.34 ± 63.47
*S. pseudintermedius* LMG 22220	22.40 ± 2.80	159.31 ± 83.91	ND

*Stationary-phase culture filtrates from 22 h at 37°C of two S. pseudintermedius isolates were analyzed by high-performance liquid chromatography-mass spectrometry (HPLC-MS). PSMs concentrations are expressed as the mean ± SD. (ND, Not detected)*.

### Cytolysis assay with synthetic PSM peptides

**Figure 3:**
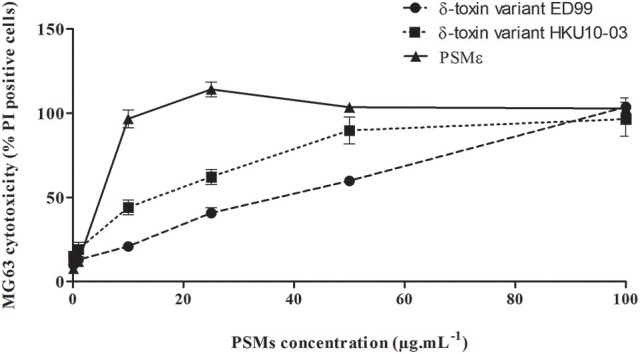
Cytolytic activity of *S. pseudintermedius* PSMs. MG63 cells were incubated with increasing concentrations (from 0.1 to 100 μg.mL^−1^) of synthetic PSMε, δ-toxin (ED99) or δ-toxin (HKU10-03) for 3 h at 37°C. Cell death was measured by staining cells with propidium iodide and detected with a fluorescence cell sorter. The percentage of cell lysis was calculated as $\%=\frac{\text{Test}-\text{Tneg}}{\text{Tpos}-\text{Tneg}}\times 100$. The values represent the means ± standard deviations derived from one experiment performed in triplicate representative of two others. NPPc, Non-professional phagocytic cells; PMNs, Polymorphonuclear neutrophils; PSM, Phenol-soluble modulins; PI, Propidium iodide.

In order to evaluate the cytotoxic effect of PSMs that we identified in the collection of *S. pseudintermedius* isolates, synthetic PSMε and δ-toxin variants covering the ranges of secreted concentrations were tested in an *in vitro* model (Figure 3). All types of PSMs tested were cytotoxic for NPPc in a dose-dependent manner. Knowing that PSMs have a receptor-independent and direct action on the lipid bilayers of cells, as expected, human PMNs were also sensitive to the different *S. pseudintermedius* PSMs (Supplementary Figure 2).

### Human serum inhibition on staphylococcal culture supernatant

**Figure 4:**
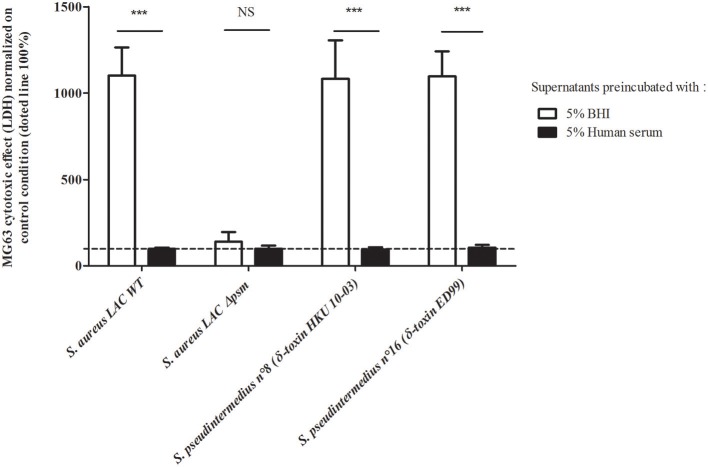
Human serum action on staphylococcal culture supernatants. MG63 cells were incubated for 4 h at 37°C with staphylococcal supernatants preincubated with 5% heat-inactivated human serum or with 5% BHI. Results are expressed as the percentages of the values obtained for the control condition “medium supplemented with BHI or human serum 5%” (100%), represented by the dotted line. Bars represent means ± standard deviations derived from three experiments performed in triplicate. The difference in the LDH concentration in staphylococcal supernatants condition compared to the uninfected control cells was evaluated using a one-tailed Mann-Whitney test with an α risk of 0.05 ^***^p < 0.001; NS, Non-significant; BHI, Brain heart infusion; LDH, Lactate dehydrogenase; NPPc, Non-professional phagocytic cells.

To confirm the hypothesis that the virulence observed on the NPPc was mediated mainly by the PSMs secreted by *S. pseudintermedius*, a serum inhibition assay was conducted firstly on *S. aureus* LAC USA300 strains and its isogenic mutant Δ*psm* (α, β, δ) (Figure 4). LDH release results were expressed as percentages of control condition (100%, medium alone). A complete abolition of cytotoxicity capacity of *S. aureus* LAC USA300 supernatant was found after the addition of human serum (LDH release - 99.02 ± 7.14%). No significant difference was observed between *S. aureus* Δ*psm* (α, β, δ) supernatant in the presence or not of human serum. This result confirmed the specific effect of human serum on PSM activity. Secondly, similarly to that found with *S. aureus* LAC USA300, the cytolytic activity of the strains *S. pseudintermedius* LMG 22220 (expressing PSMε and δ-toxin variant ED99) and *S. pseudintermedius* ST20112488 (expressing PSMε and δ-toxin variant HKU10-03) were completely abolished in presence of human serum addition (respectively, 1097.22 ± 143.84% vs. 106.71 ± 14.84%, *p* < 0.001 and 1081.94 ± 224.04% vs. 96.68 ± 11.71%, *p* < 0.001). Moreover, the cytotoxicity induced by synthetic PSMs peptides was completely inhibited in presence of inactivated human serum whereas Luk-I activity is not affected (Supplementary Figure 3). The data indicates the preponderant PSM action in the cytotoxicity of NPPc and consequently the key role of these in the virulence of *S. pseudintermedius*.

## Discussion

*S. pseudintermedius* is an opportunistic pathogen responsible for particularly severe infection both in humans and dogs. The severity of these infections has been illustrated by several clinical cases including cellulitis, purulent exudate, inflammation, and necrosis (Weese et al., 2009; Stegmann et al., 2010; Riegel et al., 2011; Mayer and Rubin, 2012; Lee et al., 2015; Darlow et al., 2017; Robb et al., 2017). Contrary to *S. aureus*, for which a panel of virulence factors have been identified and characterized in the various pathophysiological contexts, knowledge about the pathogenesis of *S. pseudintermedius* remains limited. Through *in vitro* assays, the present study allowed us to identify the key players of the high cellular virulence associated with *S. pseudintermedius* infections.

The study found that the supernatant of *S. pseudintermedius* had a strong cytotoxic effect on NPPc suggesting that this cytotoxicity is mediated, at least in part, by secreted virulence factors. Several authors have suggested that Luk-I (present in all *S. pseudintermedius* isolates tested) could be responsible for cell death induction in NPPc (Garbacz et al., 2013; Pietrocola et al., 2015). Herein, no cytotoxic activity was observed with the recombinant Luk-I toxin against NPPc over the range of concentrations tested, conversely to that found for PMN cells. The lack of Luk-I activity on NPPc led us to draw parallels with *S. aureus* leukotoxins that induce cell lysis only on immune cells harboring specific receptors. To address this question, we performed an *in vitro* assay with U937 promyelocytic cells stably transfected with C5a and CXCR2 receptors, which are respectively the target receptors of PVL and LukED leukotoxin. The results indicate that Luk-I induces cell death via the CXCR2 receptor specifically present on immune cells (PMNs, macrophages) as previously reported for leukotoxin LukED for *S. aureus*, explaining the absence of cytotoxicity observed in NPPc. This feature is likely related to the high homology between Luk-I and LukED (Spaan et al., 2017). Interestingly, this receptor, found both in humans and dogs, also shares a high homology (75%) (Hall et al., 2010).

Wang et al. have reported the contribution of a family of pro-inflammatory and cytolytic staphylococcal peptide toxins, called PSMs, in several disease manifestations (Wang et al., 2007). Moreover, Cheung et al. reported that PSMs δ, β, ε were present in the genome of *S. pseudintermedius* through *in silico* studies (Cheung et al., 2014). Herein, *psm*ε and *hld* genes, encoding for the two PSMs described in *S. pseudintermedius* able to induce cytolysis, were present in all clinical isolates (Table 1) (Otto, 2014). Analysis by HPLC-MS of δ-toxin and PSMε showed that these toxins were secreted at a high level (up to 100 μg.mL^−1^) by clinical *S. pseudintermedius* strains in *in vitro* conditions. Regarding the level of PSMs secreted *in vivo*, to the best of our knowledge, only one study has reported data on *S. aureus*; on *in vivo* samples of human pus, in which PSMs concentrations were detected up to 20 μg.mL^−1^ (Hodille et al., 2016). Using synthetic peptides, we found that all *S. pseudintermedius* PSMs studied herein are able to induce osteoblast death in a dose-dependent manner. These results confirm the cytolytic action of α-class PSMs for which it is well established in the literature that these peptides can disrupt plasma membranes independently of cell type (Wang et al., 2007; Otto, 2014). Because most α-type PSMs are sometimes too short to be annotated in genome sequences and to yield significant results in similarity analysis, *in silico* studies do not allow the detection of all α-type *psm* genes in a given genome. It would be of interest to more deeply explore the potential presence of other PSMs involved in the virulence of *S. pseudintermedius*.

The construction of isogenic mutant Δ*psm* would have been the classical way to definitively confirm that PSMs are the main factors responsible for *S. pseudintermedius* cytotoxicity on NPPc. Unfortunately, isogenic gene deletion mutants are extremely difficult to obtain in this species, which is further compounded by the multiplicity of PSMs and their functional redundancy, making it necessary to produce sequential, multiple knockouts for *psm* gene loci; such a construction was unsuccessful (data not shown). Faced with this technical limit, we chose another strategy which involved the addition of human serum that is known to be specific inhibitors of PSM action (Surewaard et al., 2012). This led to a complete loss of the cytotoxicity effect of *S. pseudintermedius* supernatant, attesting to the role of PSMs in the virulence of *S. pseudintermedius*.

**Figure 5:**
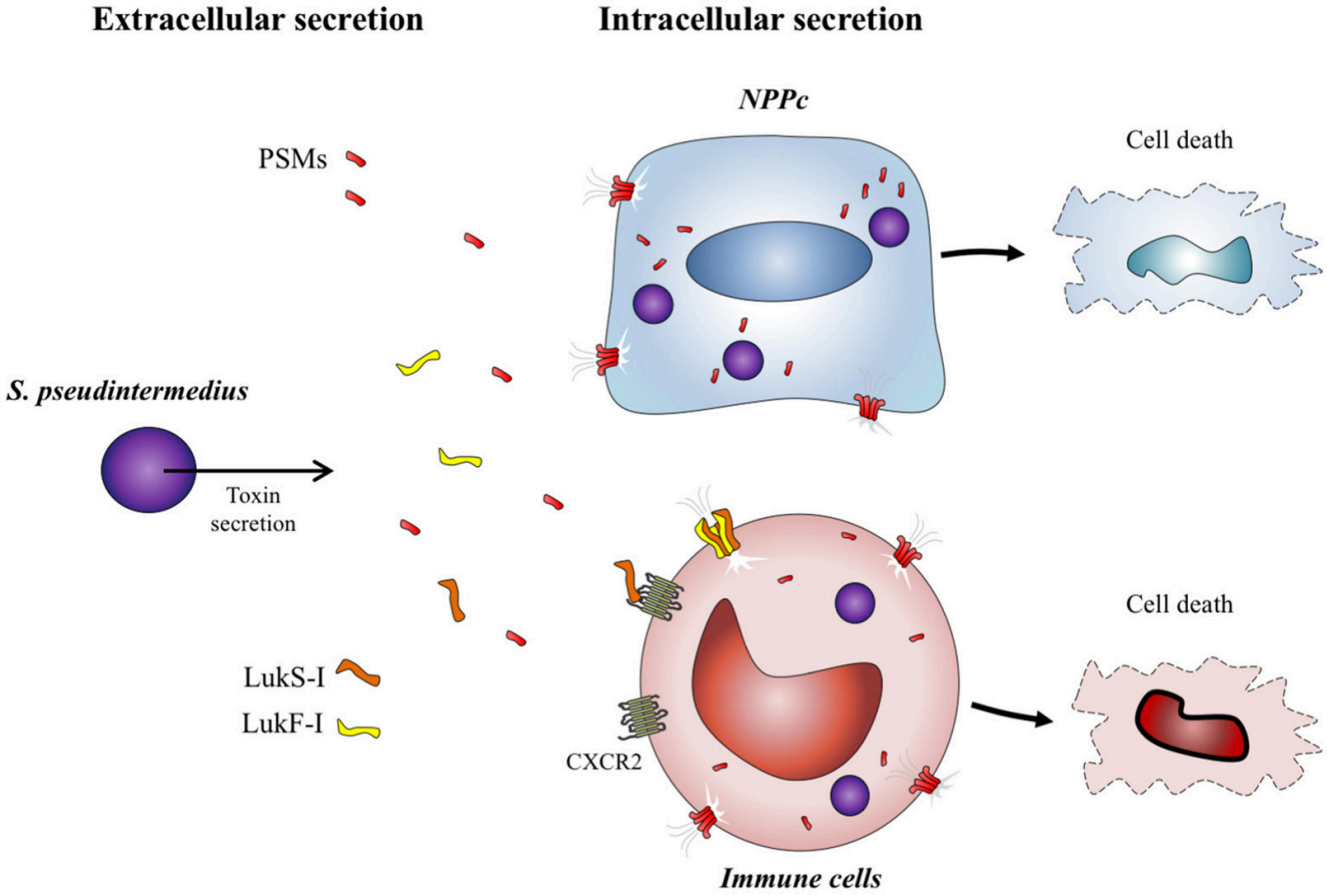
*S. pseudintermedius* toxins action on host cells. *S. pseudintermedius* can cause the necrosis of immune cells such as human polymorphonuclear neutrophils (PMNs) through the release of bicomponent leukotoxin Luk-I which recognizes the CXCR2 receptor. *S. pseudintermedius* is also able to secrete other membrane-damaging virulence factors: the phenol-soluble modulins (PSMs) which have a cytotoxic effect on both immune cells and NPPc.

The results of the present study reinforce the central role of PSMs in the virulence of staphylococci. These molecules are widespread among staphylococcal species and participate in pleiotropic action such as cytolysis (Rasigade et al., 2013; Da et al., 2017), surface colonization (Tsompanidou et al., 2013), biofilm structure and biofilm detachment (Wang et al., 2011). *S. pseudintermedius* is preferentially involved in skin and soft tissue infections with muco-purulent exudates, environments in which these amphipathic toxins retain all their active properties. In addition, *S. pseudintermedius* presents the rare capacity to internalize into host cells, therefore the intracellular compartment also constitutes a microenvironment for the accumulation of PSMs and protection from serum lipoproteins (Rasigade et al., 2013). Virulence of *S. pseudintermedius* is explained mainly by PSMs acting both in the extracellular and intracellular compartments of cells and by a selective action of Luk-I toxins acting on immune cells expressing CXCR2 receptors (Figure 5).

The present study helps elucidate bacterial pathophysiological mechanisms that could explain the severity of human and animal infections related to *S. pseudintermedius* which behaves similarly to *S. aureus*. Both harbor a variety of common virulence factors including similar/homologous MSCRAMMs, bi-component leukotoxin and PSMs, and both species are able to induce clinically severe infections. *In vitro*, they exhibit the same behavior toward human cells: they are similarly able to attack, invade and lyse human cells. Interestingly, while *S. pseudintermedius* infections are highly prevalent in pets, especially in dogs, in which it colonizes in particular mucocutaneous sites, such as the nose, mouth and anus with a carriage rate that reaches 92% in some studies, human *S. pseudintermedius* infections are scarce and classically occur after contact with animals and in presence of skin barrier defects (Allaker et al., 1992a,b; Rubin and Chirino-Trejo, 2011; Bannoehr and Guardabassi, 2012). This could be explained by the carriage rate in humans, which has been demonstrated to be very low (Paul et al., 2011; Trouillet-Assant et al., 2015). In contrast, *S. aureus* infections are very common in humans and related to preexisting nasal carriage (self-contamination) or to transmission from colonized health caregivers. This suggests that the host specificity of the two species might be related to the specific colonizing capacity of each species. Further studies deserve to be conducted to explore this differential ability and to identify the mechanisms.

## Author contributions

FL and ST-A: conceived and designed the experiments; YM and CB: performed the experiments; YM: data collection; YM, CB, PM-S, and EH: data analysis; MB, FV, AD, GL, ST-A, and FL: data interpretation; YM and ST-A: drafting manuscript; CB, EH, MB, GL, AD, ST-A, and FL: revising manuscript content; FL: takes responsibility for the integrity of the data analysis.

### Conflict of interest statement

The authors declare that the research was conducted in the absence of any commercial or financial relationships that could be construed as a potential conflict of interest.
