# Supplementary material for: Understanding the Virulence of Staphylococcus pseudintermedius: A Major Role of Pore-Forming Toxins
Source: Front Cell Infect Microbiol. 2018 Jun 28;8:221. doi: 10.3389/fcimb.2018.00221 (PMC6032551; doi:10.3389/fcimb.2018.00221)
Supplement: Supplementary file 1 [file Data_Sheet_1.DOCX]

Supplementary Material

# Understanding the virulence of *Staphylococcus pseudintermedius*:

# a major role of pore-forming toxins

Yousef Maali, Cédric Badiou, Patrícia Martins-Simões, Elisabeth Hodille, Michele Bes, François Vandenesch, Gérard Lina, Alan Diot, Frederic Laurent^*^, Sophie Trouillet-Assant

**^*^Corresponding author:** Pr. Frédéric Laurent, Centre International de Recherche en Infectiologie, INSERM U1111, CNRS UMR5308, Université de Lyon 1, ENS de Lyon, Team “Pathogenesis of staphylococcal infections”, Lyon, France.

Laboratoire de Bactériologie, Groupement Hospitalier Nord, 103 Grande Rue de la Croix-Rousse, 69004 Lyon, France.

Tel: +33 (0)4 72 07 18 37; E-mail: frederic.laurent@univ-lyon1.fr


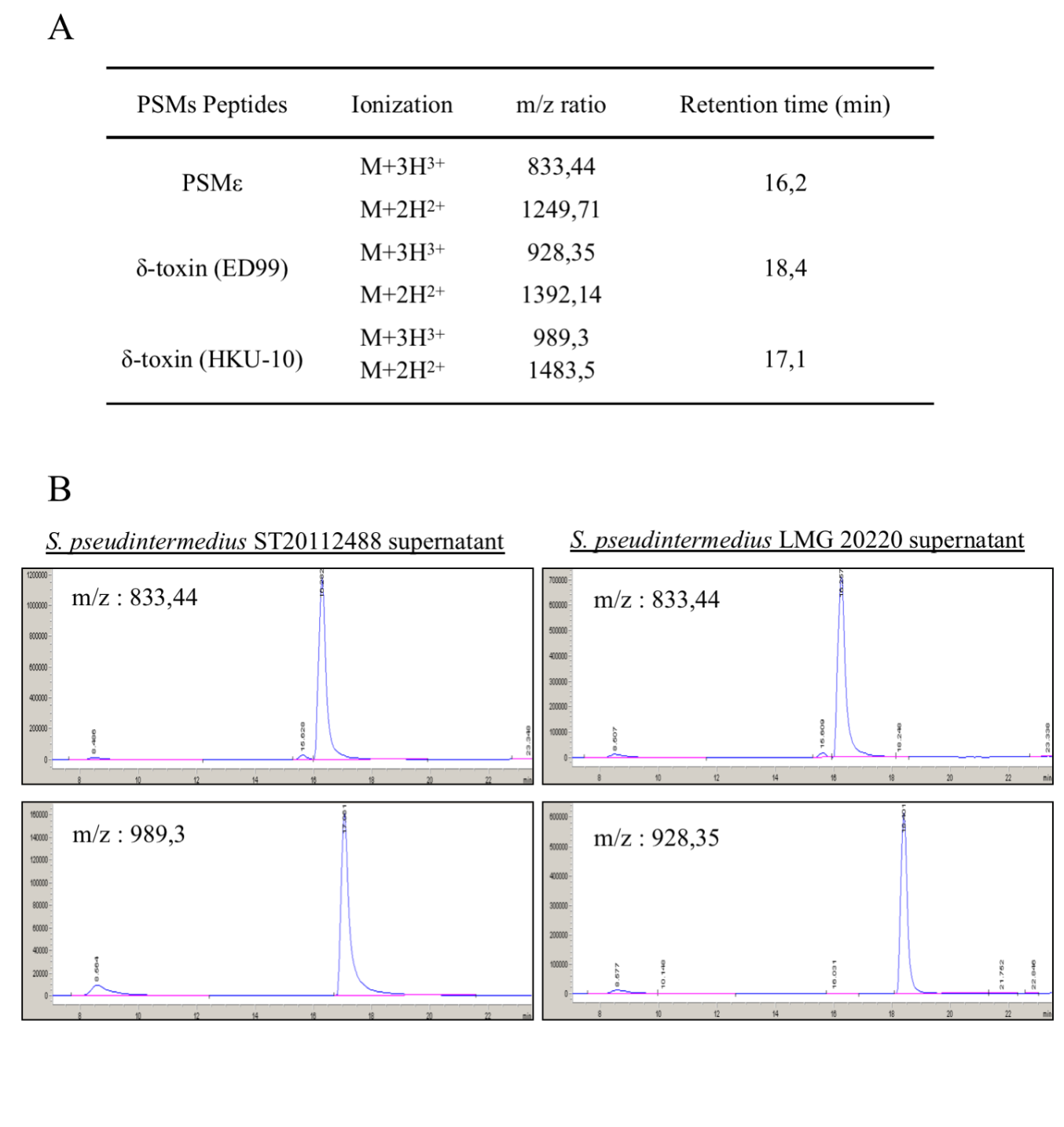


**Supplementary Figure 1:** HPLC/MS analysis of *S. pseudintermedius* culture filtrate (A) m/z ratio and retention time (min) for each PSMs screened. (B) Extracted-ion chromatogram (EIC) from stationary-phase culture filtrates of *S. pseudintermedius* subjected to HPLC/MS- targeted detection of PSMε, δ-toxin (ED99) and δ-toxin (HKU-10). X-axis represents time and Y-axis represents signal intensity.
