# Supplementary material for: Understanding the Virulence of Staphylococcus pseudintermedius: A Major Role of Pore-Forming Toxins
Source: Front Cell Infect Microbiol. 2018 Jun 28;8:221. doi: 10.3389/fcimb.2018.00221 (PMC6032551; doi:10.3389/fcimb.2018.00221)
Supplement: Supplementary file 2 [file Data_Sheet_2.DOCX]

Supplementary Material

# Understanding the virulence of *Staphylococcus pseudintermedius*:

# a major role of pore-forming toxins

Yousef Maali, Cédric Badiou, Patrícia Martins-Simões, Elisabeth Hodille, Michele Bes, François Vandenesch, Gérard Lina, Alan Diot, Frederic Laurent^*^, Sophie Trouillet-Assant

**^*^Corresponding author:** Pr. Frédéric Laurent, Centre International de Recherche en Infectiologie, INSERM U1111, CNRS UMR5308, Université de Lyon 1, ENS de Lyon, Team “Pathogenesis of staphylococcal infections”, Lyon, France.

Laboratoire de Bactériologie, Groupement Hospitalier Nord, 103 Grande Rue de la Croix-Rousse, 69004 Lyon, France.

Tel: +33 (0)4 72 07 18 37; E-mail: frederic.laurent@univ-lyon1.fr





**Supplementary Figure 2: *S. pseudintermedius* PSMs activity on human PMNs.** Human PMNs cells from 3 different donors were incubated 3h at 37°C with increasing concentrations (from 1 to 100 µg.mL-1) of synthetic PSMɛ, δ-toxin (ED99) or δ-toxin (HKU10-03). Cell death was measured by staining cells with propidium iodide (PI). The values represent the means ± standard deviations derived from three experiments. Dotted line represents the negative control without toxin. PMNs: Polymorphonuclear neutrophils; PSM: phenol-soluble modulins; PI: propidium iodide.
