# Supplementary material for: Understanding the Virulence of Staphylococcus pseudintermedius: A Major Role of Pore-Forming Toxins
Source: Front Cell Infect Microbiol. 2018 Jun 28;8:221. doi: 10.3389/fcimb.2018.00221 (PMC6032551; doi:10.3389/fcimb.2018.00221)
Supplement: Supplementary file 3 [file Data_Sheet_3.DOCX]

Supplementary Material

# Understanding the virulence of *Staphylococcus pseudintermedius*:

# a major role of pore-forming toxins

Yousef Maali, Cédric Badiou, Patrícia Martins-Simões, Elisabeth Hodille, Michele Bes, François Vandenesch, Gérard Lina, Alan Diot, Frederic Laurent^*^, Sophie Trouillet-Assant

**^*^Corresponding author:** Pr. Frédéric Laurent, Centre International de Recherche en Infectiologie, INSERM U1111, CNRS UMR5308, Université de Lyon 1, ENS de Lyon, Team “Pathogenesis of staphylococcal infections”, Lyon, France.

Laboratoire de Bactériologie, Groupement Hospitalier Nord, 103 Grande Rue de la Croix-Rousse, 69004 Lyon, France.

Tel: +33 (0)4 72 07 18 37; E-mail: frederic.laurent@univ-lyon1.fr

**

**

**Supplementary Figure 3:** Human serum action on Leukotoxin Luk-I and PSMɛ on U937 cells expressing the CXCR2 receptors.

Toxins activity was evaluated on U937 cells expressing the CXCR2 receptors preincubated with 5% heat-inactivated human serum or with 5% RPMI. Cells were incubated with Luk-I (1 µg.mL^-1^) and/or with PSMɛ (100 µg.mL^-1^) for 3 h at 37°C. Cell death was measured by staining cells with propidium iodide (PI) and detected with a fluorescence cell sorter. The percentage of cell lysis was calculated as % = $\frac{Test-Tneg}{Tpos-Tneg}$ x 100. The negative control and positive control were performed with cell buffer and lysis buffer (Triton X100 0.1%), respectively. The values represent the means ± standard deviations derived from three experiments performed in triplicate. Cytotoxicity with and without human serum was assessed using the Mann-Whitney one-tailed test with an α risk of 0.05 (*** p<0.001; NS: Not significant). U937-CXCR2: U937 cells transfected with CXCR2 receptor, RPMI: Roswell Park Memorial Institute medium, PI: propidium iodide.
