# Supplementary material for: Understanding the Virulence of Staphylococcus pseudintermedius: A Major Role of Pore-Forming Toxins
Source: Front Cell Infect Microbiol. 2018 Jun 28;8:221. doi: 10.3389/fcimb.2018.00221 (PMC6032551; doi:10.3389/fcimb.2018.00221)
Supplement: Supplementary file 4 [file Table_1.DOCX]

Supplementary Material

# Understanding the virulence of *Staphylococcus pseudintermedius*:

# a major role of pore-forming toxins

Yousef Maali, Cédric Badiou, Patrícia Martins-Simões, Elisabeth Hodille, Michele Bes, François Vandenesch, Gérard Lina, Alan Diot, Frederic Laurent^*^, Sophie Trouillet-Assant

**^*^Corresponding author:** Pr. Frédéric Laurent, Centre International de Recherche en Infectiologie, INSERM U1111, CNRS UMR5308, Université de Lyon 1, ENS de Lyon, Team “Pathogenesis of staphylococcal infections”, Lyon, France.

Laboratoire de Bactériologie, Groupement Hospitalier Nord, 103 Grande Rue de la Croix-Rousse, 69004 Lyon, France.

Tel: +33 (0)4 72 07 18 37; E-mail: frederic.laurent@univ-lyon1.fr

**Supplementary Table S1:** Conditions of gene amplification by PCR. (A) The reaction mixture for the PCR assays. (B) Sequences of the primers used for PCR. (C) Details of the protocol used for DNA amplification.

A

| **PCR mixture components** | **Volume** | **Concentration/quantity** |
| --- | --- | --- |
| Buffer | 2.5 µL | 10X |
| MgCl_2_ | 0.75 µL | 25 mM |
| dNTP (deoxynucleoside triphosphate) | 4 µL | 20 mM |
| Primer 1 | 1 µL | 10 µM |
| Primer 2 | 1 µL | 10 µM |
| Taq polymerase | 0.125 µL | 5 U/µL |
| DNA | 2 µL | - |
| H_2_O | qs 25 µL | - |

B

|  | **Primer 1 forward (5’–3’)** | **Primer 2 reverse (5’–3’)** | | **Size (bp)** |
| --- | --- | --- | --- | --- |
|  |  | |  |  |
| *hld* | CTTAGTCATTGTATTCTTCGCTCA | | TGCTACAATGGCTTCAATATCG | 270 |
| *psmɛ* | ATTTCGGCATGCACAACTG | | GCCTCAAACTCAGTCAAACGA | 230 |
| *luk*S-I | CAGGGATCCGCAAACACTATAGAAGAAATCG | | GCTTGTCGACCTATTAATTATGCCCCTTTAC | 868 |
|  |  | |  |  |
| *luk*F-I | TGTCGGATCCGCTAATCAAATTACACCTG | | GTCAGTCGACCTATTATGATGGGTTTTTTTCATC | 926 |
|  |  | |  |  |

C

| **PCR Step** | **Times and temperatures** |
| --- | --- |
| Initial denaturation | 5 min - 94°C |
| Cyclic denaturation | 30 sec - 94°C |
| Cyclic hybridization | 30 sec - 58°C x25 cycles |
| Cyclic elongation | 1 min - 72°C |
| Final elongation | 5 min - 72°C |
